# Young-Onset Gastrointestinal Adenocarcinoma Incidence and Survival Trends in the Northern Territory, Australia, with Emphasis on Indigenous Peoples

**DOI:** 10.3390/cancers14122870

**Published:** 2022-06-10

**Authors:** Mia Shepherdson, Shalem Leemaqz, Gurmeet Singh, Courtney Ryder, Shahid Ullah, Karla Canuto, Joanne P. Young, Timothy J. Price, Ross A. McKinnon, Stephen J. Pandol, Claire T. Roberts, Savio George Barreto

**Affiliations:** 1 Flinders Medical Center, Bedford Park, Adelaide, SA 5042, Australia; mia.shepherdson@sa.gov.au; 2 College of Medicine and Public Health, Flinders University, Adelaide, SA 5001, Australia; shalem.leemaqz@flinders.edu.au (S.L.); courtney.ryder@flinders.edu.au (C.R.); shahid.ullah@flinders.edu.au (S.U.); ross.mckinnon@flinders.edu.au (R.A.M.); 3 Flinders Health and Medical Research Institute, Adelaide, SA 5042, Australia; 4 Menzies School of Health Research, Darwin, NT 0810, Australia; gurmeet.singh@menzies.edu.au; 5 The George Institute for Global Health, Newtown, NSW 2042, Australia; 6 Rural and Remote Health, College of Medicine and Public Health, Flinders University, Darwin, NT 0810, Australia; karla.canuto@flinders.edu.au; 7 Department of Haematology and Oncology, The Queen Elizabeth Hospital, Woodville South, SA 5011, Australia; joanne.young@adelaide.edu.au (J.P.Y.); timothy.price@sa.gov.au (T.J.P.); 8 SAHMRI Colorectal Node, Basil Hetzel Institute, Woodville South, SA 5011, Australia; 9 Faculty of Health and Medical Sciences, University of Adelaide, Adelaide, SA 5005, Australia; 10 Division of Digestive and Liver Diseases, Cedars-Sinai Medical Center, Los Angeles, CA 90048, USA; stephen.pandol@cshs.org

**Keywords:** outcomes, morbidity, mortality, stomach, pancreas, colon, Indigenous

## Abstract

**Simple Summary:**

This study from the Australian Northern Territory’s Cancer Registry data provides evidence for a significant decrease in incidence of gastrointestinal (oesophageal, stomach, small intestine, colon, rectum, and pancreas) adenocarcinomas over the last 3 decades in individuals aged >50 years, whilst the younger (18–50 years) cohort has remained unchanged with a (non-significant) trend towards an increase. There has been a significantly improved overall survival in both age cohorts. An insight into these trends amongst Australia’s Indigenous (who constitute 31% of the territory’s population) confirms that while the incidence was significantly lower in Indigenous patients compared to non-Indigenous patients, in both age cohorts, Indigenous patients had worse survival rates. This study calls for a concerted effort aimed at investigating the existence of modifiable sociodemographic factors underlying these disturbing trends. There is a need to enhance preventative strategies, as well as to improve the delivery of cancer care and its uptake amongst Indigenous peoples.

**Abstract:**

Background and Aims: A concerning rise in incidence of young-onset cancers globally led to the examination of trends in incidence and survival of gastrointestinal (GI) adenocarcinomas in the Northern Territory (NT), Australia, over a 28-year period, with a special emphasis on Indigenous peoples. Methods: This cross-sectional analysis of a prospective longitudinal database, NT Cancer Registry (1990–2017), includes all reported cases of GI (oesophagus, gastric, small intestinal, pancreas, colon, and rectum) adenocarcinomas. Poisson regression was used to estimate incidence ratio ratios, and survival was modelled using Cox proportional hazard models separately for people aged 18–50 years and >50 years. Results: A total of 1608 cases of GI adenocarcinoma were recorded during the time of the study. While the overall incidence in people 18–50 years remained unchanged over this time (*p* = 0.51), the rate in individuals aged >50 years decreased (IRR = 0.65 (95% CI 0.56–0.75; *p* < 0.0001)). Incidence rates were significantly less in females >50 years (IRR = 0.67 95% CI 0.59–0.75; *p* < 0.0001), and their survival was significantly better (HR = 0.84 (95%CI 0.72–0.98; *p* < 0.03)) compared to males. Overall survival across all GI subsites improved in both age cohorts, especially between 2010 and 2017 (HR = 0.45 (95%CI 0.29–0.72; *p* < 0.0007) and HR = 0.64 (95%CI 0.52–0.78; *p* < 0.0001), respectively) compared to 1990–1999, driven by an improvement in survival in colonic adenocarcinoma alone, as the survival remained unchanged in other GI subsites. The incidence was significantly lower in Indigenous patients compared to non-Indigenous patients, in both age cohorts (18–50 years IRR = 0.68 95% CI 0.51–0.91; *p* < 0.009 and >50 years IRR = 0.48 95% CI 0.40–0.57; *p* < 0.0001). However, Indigenous patients had worse survival rates (18–50 years HR = 2.06 95% CI 1.36–3.11; *p* < 0.0007 and >50 years HR = 1.66 95% CI 1.32–2.08; *p* < 0.0001). Conclusions: There is a trend towards an increased incidence of young-onset GI adenocarcinomas in the NT. Young Indigenous patients have lower incidence but worse survival across all GI subsites, highlighting significant health inequities in life expectancy. Targeted, culturally safe Indigenous community-focussed programs are needed for early detection and patient-centred management of GI adenocarcinomas.

## 1. Introduction

Globally, there have been several reports of increasing incidence of early- or young-onset cancers [1,2,3,4]. Recently, we confirmed this disturbing trend over the past 28 years in South Australia by interrogating data from the South Australian Cancer Registry [5]. Early life events have long been suspected to play a role in the causation of young-onset cancers [6,7]. We proposed the Perinatal and Early Life Influences on CANcer (PELICan) hypothesis [8], providing supporting evidence from the literature, linking perinatal stressors that not only affect the epigenome, but also have an increased propensity to affect these children in their adolescent years, resulting in an increased risk of cancer. This can be further influenced by social determinants of health, which produce health inequities between populations. For example, higher cancer incidence rates have been observed in individuals living with a socioeconomic disadvantage [9,10].

Internationally, Indigenous peoples are the longest surviving civilisations on earth and continue to face significant health disparities and marginalisation across multiple social indicators (socioeconomic status, life expectancy, education) from ongoing colonisation [11,12,13,14,15]. In Australia, cancer inequities in outcomes are evident between Indigenous (Aboriginal and Torres Strait Islander) and non-Indigenous patients [16,17,18,19,20]. These disparities prompted the implementation of the National Aboriginal and Torres Strait Islander Cancer Framework in 2014 [21,22]. Given the compelling relationship between young-onset cancers and significant health inequities such as (lower) socioeconomic status, one of our aims was to explore inequity impacts in the incidence and survival of GI adenocarcinomas for Indigenous peoples in the NT. Indigenous Australians make up 3% of Australia’s population [23] but account for 31% of the NT population [24]. Understanding manifestations of inequity in young-onset cancer will provide much-needed insight into impacts on Australia’s Indigenous and provide critical understanding surrounding cancer preventative strategies (i.e., early detection), which have been created to improve the health of Indigenous Australians.

## 2. Materials and Methods

The NT Cancer Registry (NTCR) captures all NT cancer diagnosis and cancer-related deaths in accordance with the requirements of the NT Cancer Registration Act (last updated in 2011) [25], specifically, the reporting of all cancer cases to the registry. This activity includes information from treating physicians where the cause of death or patient demographics are incomplete. The registry provides a clinical epidemiological repository of cancers in the NT, to inform government initiatives, as well as established screening and prevention programs. A cross-sectional analysis, from 1 January 1990 to 31 December 2017, of prospectively collected longitudinal data was undertaken, focussing on all cases of adenocarcinoma: oesophagus, stomach, small intestine, pancreas, colon, and rectum.

Ethics approval for this study was obtained from the Aboriginal Ethics Sub-Committee (AESC) of the Human Research Ethics Committee of the NT Department of Health and Menzies School of Health Research (HREC) (HREC Reference Number: 2021-4043).

### 2.1. Selection of Cases

#### Inclusion Criteria

NT residents aged >18 years, with a pathologically confirmed diagnosis of GI adenocarcinoma; ICD-10-AM codes C15 oesophagus, C16 stomach, C17 small intestine, C25 pancreas, C18 colon (excluding C18.1), C19 rectosigmoid junction, C20 rectum. Histology codes for adenocarcinoma: ICD-10-AM 8140/2, 8140/3, 8141/3, 8143/3, 8210/2, 8210/3 and 8230/2.

The study period was categorised into 3 time periods (1990–1999, 2000–2009, 2010–2017) to reflect incidence and survivals of cancers over time. 

### 2.2. Statistical Analysis

Statistical analyses were conducted using R version 4.1.1 (R Foundation for Statistical Computing, Vienna, Austria). Patients’ characteristics were expressed as median and interquartile range (IQR) for skewed data. The Mann–Whitney U test was used to explore the significance of differences in patients’ age between two groups of patients. Proportions were presented as percentages of the respective denominator and were compared between groups using a standard Chi-square test for association with continuity correction, where appropriate.

The incidence rates were calculated by taking the total number of cases divided by the population at risk. The rates were presented per 100,000 persons over 3 time periods for age groups 18–50 years and >50 years, for each sex, Indigenous status and cancer primary sites. Mid-interval population references for each time period were used as the denominator in the calculation of incidence rates. A Poisson regression model was applied to examine the incidence rates between groups of the above characteristics. The estimates were calculated using the likelihood ratio method and were expressed as incidence rate ratios (IRRs) from the Poisson model. The Poisson regression model was also used to calculate the average annual percentage change.

Survival was measured from the date of cancer diagnosis to the date of death, and individuals were censored at date of loss to follow-up or census date. The census date was assigned on 31 December 2017. The NTCR data are linked to the Births, Deaths, and Marriage data annually. Complete data were obtained on survival days, age of diagnosis, date of death, sex, primary sites, and Indigenous status. Cox proportional hazard models were applied to examine the survival outcomes. Sex, primary sites, Indigenous status, and cohort era were used to explore the risk of death between two cohorts (18–50 years and >50 years). The estimates were calculated using the likelihood ratio method and were expressed as hazard ratios (HRs); the lower the HR, the longer the survival. Proportional hazard assumption was tested by a log–log plot of survival and Schoenfeld Residuals. Survival curves for patient survival were evaluated by standard Kaplan–Meier survival curves, and patient cohorts were compared by log-rank test. The two-sided test was performed for all analyses, 95% confidence intervals were reported, and the level of significance was set at 5%.

## 3. Results

### 3.1. Patient Data and Overall Incidence and Survival Trends

A total of 1608 patients were diagnosed with oesophageal, stomach, small intestinal, pancreatic, or colorectal adenocarcinoma in the NT between 1990 and 2017 (298, 18.5% patients aged 18–50 years and 1310, 81.5% patients aged >50 years) (Table 1). Adenocarcinoma of the colon was the most commonly reported subsite involved in both age cohorts (27.2/100,000 for individuals aged 18–50 years and 900.3/100,000 for those aged >50 years).

**Figure 1:**
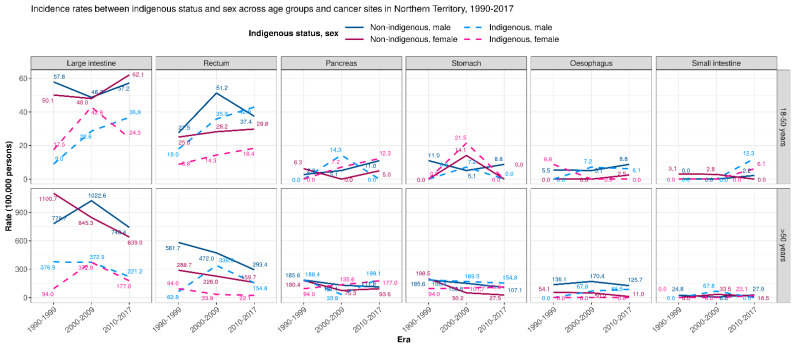
Trend in incidence rates by Indigenous status, sex, and era between two age groups across cancer sites, 1990–2017 (*n* = 1608).

Age, in itself, was a contributing factor for incidence, with a higher increment per year of age for individuals aged 18–50 years (IRR = 1.15 (95%CI 1.13–1.17; *p* < 0.0001)) as compared to those >50 years (IRR = 1.06 (95%CI 1.06–1.07; *p* < 0.0001)) (Table 2). The overall cancer incidence rates varied by sex for the >50 years cohort (131.1/100,000 for females and 196.9/100,000 for males >50 years) with IRR = 0.67 (95%CI 0.59–0.75; *p* < 0.0001). There was a significantly lower incidence of adenocarcinomas in every subsite for females in the >50 years cohort, except for pancreatic adenocarcinoma (Figure 1). In the 18–50 years cohort, no significant difference in incidence, by sex of the patient, was noted either in the overall cohort (4.4/100,000 for females and 5.4/100,000 for males aged 18–50 years; *p* = 0.09), or by organ subsite in the 18–50 years cohort (Table 2, Figure 1 and Figure S1).

There was a significantly improved survival across all GI adenocarcinoma subsites over the 3 time cohorts (HR = 0.45 (95%CI 0.29–0.72; *p* = 0.0007)) for those aged 18–50 years and 0.64 (95%CI 0.52–0.78; *p* < 0.0001) for those >50 years, respectively, between 2010 and 2017 compared to years 1990 to 1999. Here also, females >50 years had a significantly improved survival compared to their male counterparts (HR = 0.84 (95%CI 0.72–0.98; *p* = 0.03)). No significant difference in survival was noted amongst females in the 18–50 years cohort (HR = 0.88 (95%CI 0.62–1.27; *p* = 0.5)).

**Figure 2:**
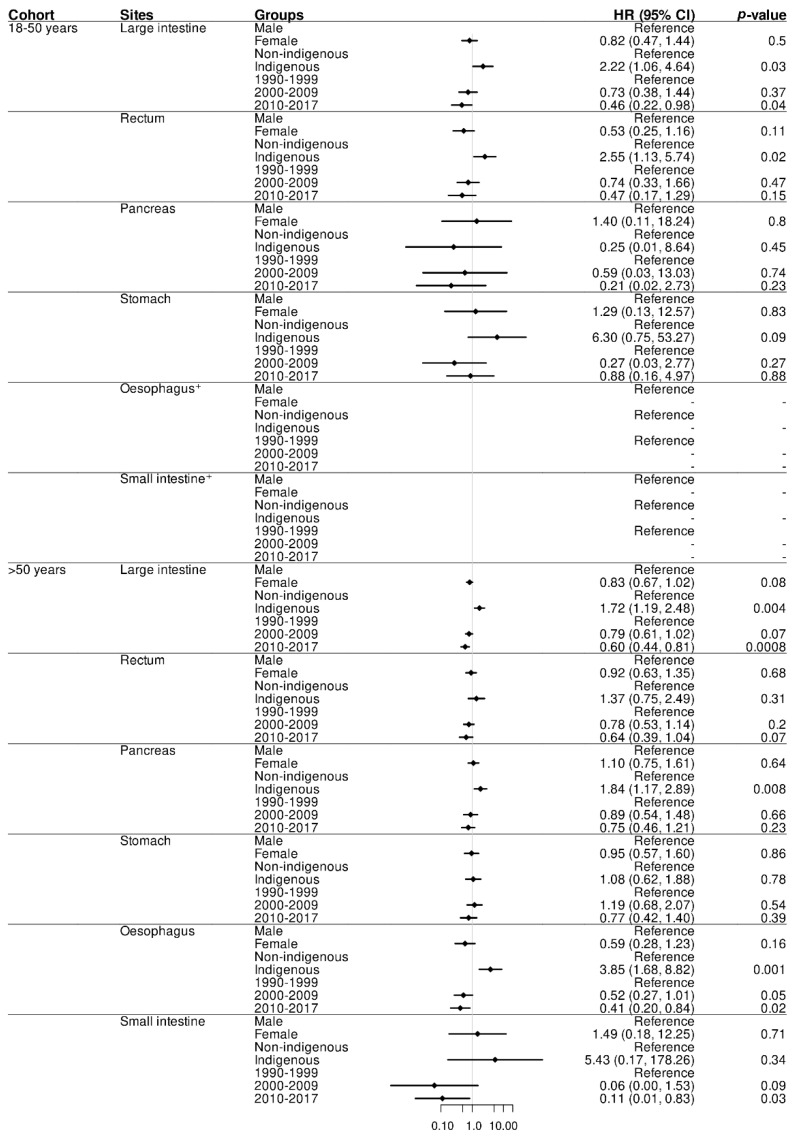
Hazard ratios (HR) and 95% CI (Cox proportional hazard model) for Indigenous status, sex, and era by primary sites between two age groups (*n* = 1608). ^+^ Reliable estimates of HR (95% CI) cannot be obtained for Oesophagus and Small intestine in 18–50 years due to small sample size.

The improvement in survival amongst the 18–50 years cohort was due only to a significantly improved survival for colonic adenocarcinoma in the last time period (HR = 0.46 (95%CI 0.22–0.98; *p* = 0.04)). On the contrary, in the >50 years cohort, the significantly improved survival was driven by improvements in survival in colonic (HR = 0.60 (95%CI 0.44–0.81; *p* = 0.0008)), oesophageal (HR = 0.41 (95%CI 0.20–0.84; *p* = 0.02)), and small intestinal (HR = 0.11 (95%CI 0.01–0.83; *p* = 0.03)) adenocarcinomas over the last time period (Figure 2).

#### 3.1.1. GI adenocarcinomas Trends across Populations

The overall incidence rate for GI adenocarcinomas in individuals aged 18–50 years in the NT remained unchanged over the last three time periods from 1990–1999 to 2010–2017 (4.7/100,000 to 5.1/100,000), and the rates amongst individuals aged >50 years decreased over the same time period from 189.5/100,000 to 122.9/100,000 (IRR = 0.65 (95%CI 0.56–0.75); *p* < 0.0001) (Table 2). Cancer incidence rates for Indigenous peoples aged 18–50 years were 4.0/100,000 and 110.7/100,000 for >50 years of ages, which was significantly lower compared to non-Indigenous peoples, 5.9/100,000 (18–50 years) with IRR = 0.68 (95%CI 0.51–0.91) (*p* = 0.009) and 233/100,000 (>50 years) with IRR = 0.48 (95%CI 0.40–0.57) (*p* < 0.0001).

**Figure 3:**
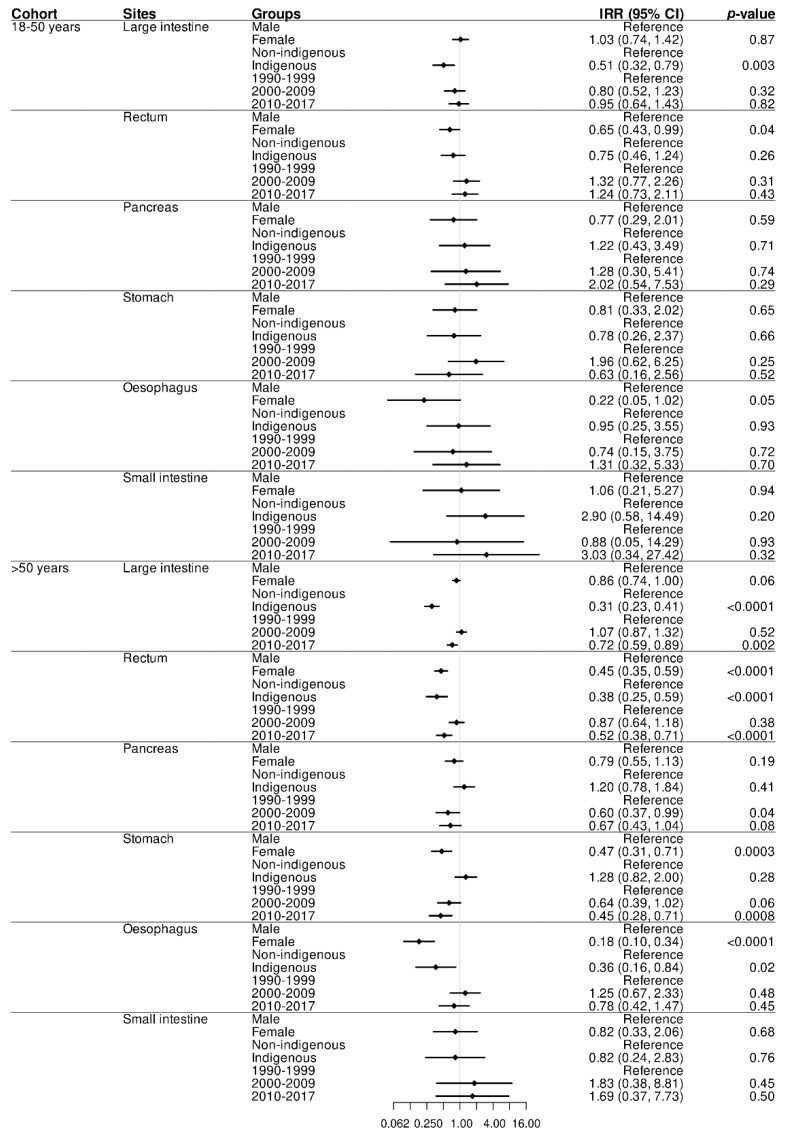
Incidence rate ratios (IRR) and 95% CI (Poisson regression model) for Indigenous status, sex, and era by primary sites between two age groups (*n* = 1608).

The reduced overall incidence of GI adenocarcinomas noted amongst Indigenous peoples in both age cohorts was largely influenced by the significantly lower incidence of colonic adenocarcinomas in the 18–50 years cohort (IRR = 0.51 (95%CI 0.32–0.79, *p* = 0.003)), and colonic and rectal adenocarcinomas in the >50 years cohort (IRR = 0.31 (95%CI 0.23–0.41, *p* < 0.0001); and (IRR = 0.38 (95%CI 0.25–0.59, *p* < 0.0001), respectively) (Figure 3 and Supplementary Table S2).

Rates of GI adenocarcinomas increased per year in males 18–50 years, with an estimated average annual percentage change (AAPC) of 2.23% (95%CI 0.32–4.18; *p* = 0.02). There was also an increasing trend for both non-Indigenous and Indigenous populations in 18–50 years, with the Indigenous population having a higher AAPC (AAPC 1.88 (95%CI 0.28–3.51; *p* = 0.02) for non-Indigenous and AAPC 3.70 (95%CI 0.24–7.28; *p* = 0.04)) (Supplementary Table S1).

#### 3.1.2. Survival by Time Trends and Site in Indigenous Compared to Non-Indigenous Peoples

Survival estimates from Kaplan–Meier plots revealed the greatest median survivals for rectal adenocarcinoma in 18–50 years (3.98 years) and colonic adenocarcinoma in >50 years (4.17 years) (Table 3). Using colonic adenocarcinoma as the reference, survival following pancreatic and stomach adenocarcinoma were significantly lower for those aged 18–50 years (HR = 2.30 (95%CI 1.19–4.46; *p* < 0.01) and HR = 2.73 (95%CI 1.49–5.02; *p* < 0.001), respectively). For those aged >50 years, survival following pancreatic (HR = 5.76 (95%CI 4.58–7.24; *p* < 0.0001)), stomach (HR = 2.91 (95%CI 2.28–3.70; *p* < 0.0001)), oesophageal (HR = 3.29 (95%CI 2.51–4.30; *p* < 0.0001)), and small intestinal (HR = 2.01 (95%CI 1.10–3.68; *p* < 0.02)) adenocarcinoma was significantly lower compared to the reference (Table 4 and Supplementary Figure S2).

Survival, following GI adenocarcinomas, was significantly lower amongst Indigenous peoples as compared to non-Indigenous peoples in both age cohorts over the study period (HR = 2.06 (95%CI 1.36–3.11; *p* < 0.0007) for the 18–50 years cohort and HR = 1.66 (95%CI 1.32–2.08; *p* < 0.0001) for the >50 years cohort, respectively) (Table 4 and Supplementary Figure S2). The significantly reduced survival for Indigenous peoples was largely the effect of colonic and rectal adenocarcinoma in those aged 18–50 years (HR = 2.22 (95%CI 1.06–4.64; *p* < 0.03) and HR = 2.55 (95%CI 1.13–5.74; *p* < 0.02), respectively). For Indigenous people >50 years, reduced survival was largely the effect of colonic (HR = 01.72 (95%CI 1.19–2.48; *p* < 0.004)), pancreatic (HR = 1.84 (95%CI 1.17–2.89; *p* < 0.008)), and oesophageal (HR = 3.85 (95%CI 1.68–8.82; *p* < 0.001)) adenocarcinomas (Supplementary Table S3).

## 4. Discussion

Our research shares the incidence and survival of GI adenocarcinomas in NT residents over a 28-year period. The incidence of young-onset cancers (18–50 years) marginally, but not statistically significantly, increased (by 10%), while the incidence in the >50 years cohort significantly reduced. Male sex was associated with poorer prognosis in older age (>50 year), whereas young-onset cancers were non-discriminatory. Poorer survival outcomes were observed in Indigenous populations despite an overall lower incidence of GI adenocarcinomas, compared to non-Indigenous individuals. This is a novel finding for young-onset cancers.

Lifestyle and environmental factors such as obesity, diet, smoking, and alcohol exposure are well-documented risk factors for young-onset GI cancers [26,27,28]. The NT records the highest percentage of both daily smokers (20%) and alcohol consumers who exceed the recommended number of daily standard drinks (21.4%), compared to other states and territories in Australia. The association between rising smoking rates and the rise in tobacco-related cancers was reported in the NT nearly a decade ago [29]. Metabolic syndrome and its contributing factors of poor diet and sedentary lifestyle have been linked to early-onset solid-organ tumours [27,28]. Liu et al. [28] demonstrated a risk ratio of 1.2 for every 5-unit increment in body mass index (BMI) in young adults diagnosed with colorectal cancer. Over the past two decades, we have seen an increase in both obesity and young-onset cancers in the Australian population [30]. This suggests a temporal association of metabolic syndrome serving as a contributory factor to the rise in young-onset cancers. However, it does not explain the stable rate of late-onset cancers despite an increase in BMI in this age group, too. Although the incidence of GI adenocarcinomas is on the rise, improved survival has been demonstrated in our young-onset cohort. This finding is in contrast to our observations in young-onset adenocarcinomas in South Australia (where the overall survival across all subsites has remained unchanged) [5]. This trend has been attributed to the significantly improved survival in colonic adenocarcinoma, especially in the last time period. Improved survival in colonic adenocarcinoma may be credited to increasing awareness of young-onset cancers or timely access to imaging and colonoscopy. Additionally, the cancer surveillance programmes (colon, breast, and cervical) are incorporated as part of primary health care and specialist outreach services in the NT. Colon cancer is also likely to present earlier than stomach and pancreatic cancer, thus allowing for a variety of treatment options with less disease burden [31]. Improved colon cancer survival is unlikely related to screening in patients with a positive family history, as young-onset colorectal cancer is more commonly diagnosed in patients without a family history [32]. The lack of hereditary predisposition has also been noted in young-onset gastric and pancreatic cancers, suggesting young-onset cancers are the result of an alternate carcinogenic pathway [33,34,35]. For this reason, simply reducing the age of screening based on family history will likely increase the burden on the health care system without improving young-onset cancer survival significantly. However, a recent modelling study found that reducing the age for Aboriginal and Torres Strait Islander peoples would be cost-effective and save more lives [36]. Additionally, investing resources into studying the cause of increased young-onset GI cancers may be more economical and beneficial for society in the long term.

Male gender has been consistently associated with greater incidence and poorer survival in GI cancers [37,38]. The Australian Bureau of Statistics reports that adult males are more likely to be obese and partake in higher rates of smoking and alcohol consumption compared to females [30,39,40]. It is therefore not surprising older males are at higher risk of developing and dying from GI cancers. Perhaps the most pertinent finding when analysing sex in our study was that the young-onset GI cancers did not discriminate between male and female patients. This may be partially explained by the PELICan hypothesis [8] as exposure to perinatal stressors and carcinogens in utero would be equal amongst male and female foetuses. Unborn males would not be at an increased risk of GI cancers compared to females as smoking, obesity, and alcohol are male-dominated risk factors experienced later in life. The PELICan hypothesis may also explain how the incidence of GI cancers was stable for the >50 years cohort but increasing for the 18–50 years cohort. Not only has obesity increased in the adult population, but also in the pregnant population, where currently 1 in 5 pregnant women are obese [41,42]. Obesity in pregnancy with its associated perinatal complications [43] may result in stress and/or inflammation-induced epigenetic changes in the foetus predisposing to obesity and young-onset cancer.

The impact of colonisation globally continues to impact the socioeconomic and health status and life expectancy for Indigenous populations as compared to non-Indigenous populations. Social determinants and lower socioeconomic status have been consistently associated with poor cancer survival [44,45]. In 2006, Anderson et al. [46] alarmingly reported that the life expectancy of Aboriginal and Torres Strait Islander peoples was 20 years lower than that for the total Australian population. The recent statistics from the Australian Institute of Health and Welfare [47] show improvements, but not parity. In 2015–2017, life expectancy at birth for Indigenous Australians was estimated to be 71.6 years for males and 75.6 years for females. In comparison, over the same period, life expectancy at birth for non-Indigenous Australians was 80.2 years for males and 83.4 years for females. Cancer survival was no exception to this health discrepancy, which prompted the Australian government to implement the National Aboriginal and Torres Strait Islander Cancer Framework in 2014 [21,22,48]. The results of our study are consistent with the literature whereby, despite a lower incidence of cancer, Indigenous patients had a significantly reduced survival in both age cohorts. These outcomes concur in part with research by Condon et al. [17], who also demonstrated poorer cancer survival amongst Indigenous peoples in the NT, with diagnoses at an advanced stage of the cancer being a contributing factor. Additionally, a previous study from South Australia has flagged issues around availability and access to surgical and systemic treatments amongst Aboriginal cancer patients compared to non-Aboriginal South Australian patients, which further complicated the disadvantages associated with geographic remoteness and advanced stage of disease at diagnosis, compounded by the presence of associated comorbid conditions [49]. In the present study, owing to the lack of stage-specific data, we are unable to comment on whether a delayed diagnosis was responsible for the poorer survival observed. Certainly, the significant health inequities that impact Indigenous Australians with ongoing marginalisation would be contributors to this trend [16]. Distance and access to tertiary health settings have been shown to contribute to later presentation and more advanced disease in Indigenous populations, along with health professionals in rural and remote settings being trained as generalists [18,50,51]. Not only is remoteness a likely impediment to regular, and timely, access to health care and poor overall cancer survival [52], but cultural marginalisation and access to culturally safe and responsive healthcare is also a compounding factor contributing to delayed disease presentations or delay in timely treatment [16,53]. Whilst underrepresentation of Indigenous status within registries remains an area of global concern [12,20], in the NT, there is good ascertainment of Indigenous status, as evidenced by no missing data in the NTCR data analysed in this study.

This study successfully reported the incidence and survival of GI adenocarcinoma in the NT and evaluated the influence of Indigenous status, age, and primary tumour location. Data were collected from the reputable NTCR. The use of three distinct time periods was effective in tracking changes in incidence and survival over time and allowed for a quick comparison to our previous South Australian study. The study could be further improved by data linkage, including data pertaining to patient socioeconomic status and premorbid baseline such as postcode, BMI, alcohol intake, and smoking status. Data linkage with national administrative and clinical datasets (e.g., Pharmaceutical Benefits Scheme/PBS and Medicare Benefits Schedule/MBS) will help provide pertinent information on costs incurred with the management of these cancers. Information on timely access to cancer treatment such as chemoradiotherapy or surgical management may help explain survival outcomes. Data stratification resulted in small cohort sample sizes, which limited the statistical power and ability to conduct an in-depth analysis on the significance of primary tumour location. Further studies with an increased sample size will likely yield information on the behaviour of different gastrointestinal cancers, which could influence current screening regimes. In order to capture data that appropriately record factors impacting on patient outcomes, data registries should engage with Aboriginal and Torres Strait Islander health bodies to broaden coding, to capture community needs, and work towards data sovereignty. Data that recognise and respond to the health and well-being concepts and needs of Australia’s First Peoples constitutes a step towards data sovereignty for Aboriginal and Torres Strait Islander communities [54,55]. An exemplar of this includes the Footprints in Time Study, with Indigenous leadership, oversight, and a focus on positive strength-based data collection and reporting [56].

## 5. Conclusions

Our study uniquely compares the incidence and survival in young-onset GI adenocarcinomas between Indigenous and non-Indigenous NT residents. This study demonstrates that not only are young-onset GI cancers increasing for residents aged 18–50 years, but there is a significant and disturbing trend of lower incidence but poorer survival for Indigenous residents of any age. Lifestyle and environmental factors during the perinatal period and into early adulthood are likely contributors to these phenomena; however, more research is imperative to identify at-risk cohorts.

## Figures and Tables

**Table 1:** Patient’s characteristics, era, and primary sites of cancer between two age groups (*n* = 1608).

	(18–50 Years)		(>50 Years)		
	*n* = 298 (18.5%)		*n* = 1310 (81.5%)		
	*n*	%	*n*	%	*p*-Value
Age (years): median (IQR)	45	(39–49)	64	(58–72)	<0.0001
Sex					0.07
Male	172	57.7	834	63.7	
Female	126	42.3	476	36.3	
Indigenous					<0.0001
Non-Indigenous	240	80.5	1179	90.0	
Indigenous	58	19.5	131	10.0	
Era					0.4
1990–1999	72	24.2	275	21.0	
2000–2009	103	34.6	501	38.2	
2010–2017	123	41.3	534	40.8	
Primary site					0.0006
Large intestine (excl. Appendix)	147	49.3	704	53.7	
Rectum	97	32.6	276	21.1	
Pancreas	17	5.7	122	9.3	
Stomach	19	6.4	108	8.2	
Oesophagus	12	4.0	81	6.2	
Small intestine	6	2.0	19	1.5	

Note. Number and percentages are reported unless stated otherwise; IQR, interquartile range. *P*-values are based on Mann–Whitney U test for medians and Chi-square test for proportions.

**Table 2:** Incidence rates (IR) and incidence rate ratios (IRR) for age, sex, era, and cancer sites between two age groups (*n* = 1608).

	(18–50 Years)			(>50 Years)		
	*n* = 298			*n* = 1310		
	* IR (95% CI)	IRR (95% CI)	*p*-Value	* IR (95% CI)	IRR (95% CI)	*p*-Value
Overall	95.64 (85.09, 107.14)			1321.70 (1251.09, 1395.26)		
Age (years)	-	1.15 (1.13, 1.17)	<0.0001	-	1.06 (1.06, 1.07)	<0.0001
Sex						
Male	5.39 (4.09, 7.10)	Reference	-	196.86 (172.34, 224.87)	Reference	-
Female	4.41 (3.31, 5.87)	0.82 (0.65, 1.03)	0.09	131.05 (113.74, 151.01)	0.67 (0.59, 0.75)	<0.0001
Indigenous						
Non-Indigenous	5.91 (4.61, 7.56)	Reference	-	232.99 (209.68, 258.88)	Reference	-
Indigenous	4.02 (2.87, 5.62)	0.68 (0.51, 0.91)	0.009	110.73 (91.44, 134.09)	0.48 (0.40, 0.57)	<0.0001
Era						
1990–1999	4.66 (3.40, 6.38)	Reference	-	189.49 (161.17, 222.79)	Reference	-
2000–2009	4.83 (3.56, 6.57)	1.04 (0.77, 1.41)	0.81	177.91 (154.23, 205.23)	0.94 (0.81, 1.09)	0.40
2010–2017	5.14 (3.82, 6.90)	1.10 (0.82, 1.48)	0.51	122.92 (106.80, 141.48)	0.65 (0.56, 0.75)	<0.0001
Cancer site						
Large intestine	27.22 (21.68, 34.18)	-	-	900.31 (807.56, 1003.71)	-	-
Rectum	17.96 (13.91, 23.19)			351.68 (304.95, 405.58)		
Pancreas	3.15 (1.91, 5.20)	-	-	156.02 (128.44, 189.53)	-	-
Stomach	3.52 (2.18, 5.67)	-	-	138.12 (112.54, 169.50)	-	-
Oesophagus	2.22 (1.23, 4.00)	-	-	103.59 (82.15, 130.63)	-	-
Small intestine	1.11 (0.49, 2.51)	-	-	24.30 (15.39, 38.36)	-	-

* IR is incidence per 100,000 Northern Territory residents. IRs were not reported for age, and IRRs were not reported for cancer sites.

**Table 3:** Median survival times (years) for primary sites of cancer between two age groups (*n* = 1608).

Cancer Sites	(18–50 Years)	(>50 Years)
	*n* = 298 (18.5%)	*n* = 1310 (81.5%)
	Median (95% CI)	Median (95% CI)
Overall	3.37 (1.59–8.66)	2.91 (1.24–7.46)
Large intestine	3.75 (1.79–9.97)	4.17 (1.75–8.45)
Rectum	3.98 (1.90–9.62)	4.06 (1.67–9.03)
Pancreas	1.59 (0.97–5.59)	0.98 (0.56–1.49)
Stomach	1.51 (1.12–2.79)	1.55 (0.85–2.81)
Oesophagus	2.19 (1.56–3.56)	1.64 (1.06–2.57)
Small intestine	1.73 (0.80–4.19)	1.86 (0.82–4.98)

**Table 4:** Hazard ratios (HR) and 95% CI (Cox proportional hazard model) for age, sex, era, and cancer sites between two age groups (*n* = 1608).

	(18–50 Years)		(>50 Years)	
	*n* = 298		*n* = 1310	
	HR (95% CI)	*p*-Value	HR (95% CI)	*p*-Value
Age (years)	1.02 (1.00, 1.05)	0.07	1.04 (1.03, 1.05)	<0.0001
Sex				
Male	Reference	-	Reference	-
Female	0.88 (0.62, 1.27)	0.5	0.84 (0.72, 0.98)	0.03
Indigenous				
Non-Indigenous	Reference	-	Reference	-
Indigenous	2.06 (1.36, 3.11)	0.0007	1.66 (1.32, 2.08)	<0.0001
Era				
1990–1999	Reference	-	Reference	-
2000–2009	0.63 (0.42, 0.97)	0.03	0.81 (0.68, 0.97)	0.02
2010–2017	0.45 (0.29, 0.72)	0.0007	0.64 (0.52, 0.78)	<0.0001
Cancer site				
Large intestine	Reference		Reference	
Rectum	0.94 (0.63, 1.42)	0.78	1.01 (0.82, 1.23)	0.95
Pancreas	2.30 (1.19, 4.46)	0.01	5.76 (4.58, 7.24)	<0.0001
Stomach	2.73 (1.49, 5.02)	0.001	2.91 (2.28, 3.70)	<0.0001
Oesophagus	1.87 (0.88, 3.98)	0.1	3.29 (2.51, 4.30)	<0.0001
Small intestine	1.88 (0.59, 6.02)	0.29	2.01 (1.10, 3.68)	0.02

## Data Availability

Authors are unable to provide this data owing to the Ethics approval being granted on the premise that the (Northern Territory Cancer Registry) data will not be released to a third party.

## Supplementary Materials

The following supporting information can be downloaded at: https://www.mdpi.com/article/10.3390/cancers14122870/s1. Table S1: Average annual percentage change, AAPC, (Poisson Regression Model) for gender, Indigenous status and primary sites of cancer between two age groups (*n* = 1608); Table S2: Incidence rate ratios (IRR) and 95% CI (Poisson regression model) for sex, Indigenous status and era by primary sites between two age groups (*n* = 1608); Table S3: Hazard ratios (HR) and 95% CI (Cox Proportional hazard model) for sex, Indigenous status and era by primary sites between two age groups (*n* = 1608); Supplementary Figure S1: Trend in incidence rates by sex, Indigenous status and era between two age groups across cancer sites 1990–2017 (n = 1608); Figure S2: Kaplan-Meier survival curves for sex, era, Indigenous status and primary sites between age groups.
