# Supplementary material for: Young-Onset Gastrointestinal Adenocarcinoma Incidence and Survival Trends in the Northern Territory, Australia, with Emphasis on Indigenous Peoples
Source: Cancers (Basel). 2022 Jun 10;14(12):2870. doi: 10.3390/cancers14122870 (PMC9220984; doi:10.3390/cancers14122870)
Supplement: Supplementary file 1 [file cancers-14-02870-s001.zip › cancers-1714562-supplementary.pdf]

Table S1. Average annual percentage change, AAPC, (Poisson Regression Model) for gender, indigenous status and primary sites of cancer between two age groups (*n*=1,308).

	(18-50 Years) *n*=298		(>50 Years) *n*=1310	
	**AAPC (95% CI)**	***p*-Value**	**AAPC (95% CI)**	***p*-Value**
Sex				
Male	2.23 (0.32, 4.18)	0.02	0.26 (-0.64, 1.16)	0.6
Female	2.07 (-0.17, 4.36)	0.07	-0.28 (-1.46, 0.92	0.6
Indigenous status				
Non-indigenous	1.88 (0.28, 3.51)	0.02	-0.18 (-0.94, 0.58)	0.6
Indigenous	3.70 (0.24, 7.28)	0.04	1.63 (-0.66, 3.97)	0.2
Cancer site				
Large intestine	1.62 (-0.43, 3.71)	0.1	0.50 (-0.49, 1.50)	0.3
Rectum	2.60 (0.04, 5.23)	0.047	-1.01 (-2.53, 0.53)	0.2
Pancreas	5.70 (-0.86, 12.69)	0.09	0.41 (-1.94, 2.82)	0.7
Stomach	-1.77 (-7.09, 3.85)	0.5	-1.96 (-4.33, 0.46)	0.1
Oesophagus	3.66 (-3.65, 11.52)	0.3	0.65 (-2.27, 3.66)	0.7
Small intestine	10.83 (-2.11, 25.48)	0.1	3.35 (-3.03, 10.15)	0.3

Table S2. Incidence rate ratios (IRR) and 95% CI (Poisson regression model) for sex, indigenous status and era by primary sites between two age groups (*n*=1,608).

	(18-50 Years) *n*=298		(>50 Years) *n*=1310	
	**IRR (95% CI)**	***p*-Value**	**IRR (95% CI)**	***p*-Value**
Large intestine				
Sex				
Male	Reference	-	Reference	-
Female	1.03 (0.74, 1.42)	0.87	0.86 (0.74, 1.00)	0.06
Indigenous status				
Non-indigenous	Reference	-	Reference	-
Indigenous	0.51 (0.32, 0.79)	0.003	0.31 (0.23, 0.41)	<0.0001
Era				
1990-1999	Reference	-	Reference	-
2000-2009	0.80 (0.52, 1.23)	0.32	1.07 (0.87, 1.32)	0.52
2010-2017	0.95 (0.64, 1.43)	0.82	0.72 (0.59, 0.89)	0.002
Rectum				
Sex				
Male	Reference	-	Reference	-
Female	0.65 (0.43, 0.99)	0.04	0.45 (0.35, 0.59)	<0.0001
Indigenous status				
Non-indigenous	Reference	-	Reference	-
Indigenous	0.75 (0.46, 1.24)	0.26	0.38 (0.25, 0.59)	<0.0001
Era				
1990-1999	Reference	-	Reference	-
2000-2009	1.32 (0.77, 2.26)	0.31	0.87 (0.64, 1.18)	0.38
2010-2017	1.24 (0.73, 2.11)	0.43	0.52 (0.38, 0.71)	<0.0001
Pancreas				
Sex				
Male	Reference	-	Reference	-
Female	0.77 (0.29, 2.01)	0.59	0.79 (0.55, 1.13)	0.19
Indigenous status				
Non-indigenous	Reference	-	Reference	-
Indigenous	1.22 (0.43, 3.49)	0.71	1.20 (0.78, 1.84)	0.41
Era				
1990-1999	Reference	-	Reference	-
2000-2009	1.28 (0.30, 5.41)	0.74	0.60 (0.37, 0.99)	0.04
2010-2017	2.02 (0.54, 7.53)	0.29	0.67 (0.43, 1.04)	0.08
Stomach				
Sex				
Male	Reference	-	Reference	-

	(18-50 Years)		(>50 Years)	
	n=298		*n*=1310	
	**IRR (95% CI)**	***p*-Value**	**IRR (95% CI)**	***p*-Value**
Female	0.81 (0.33, 2.02)	0.65	0.47 (0.31, 0.71)	0.0003
Indigenous status				
Non-indigenous	Reference	-	Reference	-
Indigenous	0.78 (0.26, 2.37)	0.66	1.28 (0.82, 2.00)	0.28
Era				
1990-1999	Reference	-	Reference	-
2000-2009	1.96 (0.62, 6.25)	0.25	0.64 (0.39, 1.02)	0.06
2010-2017	0.63 (0.16, 2.56)	0.52	0.45 (0.28, 0.71)	0.0008
Osophagus				
Sex				
Male	Reference	-	Reference	-
Female	0.22 (0.05, 1.02)	0.05	0.18 (0.10, 0.34)	<0.0001
Indigenous status				
Non-indigenous	Reference	-	Reference	-
Indigenous	0.95 (0.25, 3.55)	0.93	0.36 (0.16, 0.84)	0.02
Era				
1990-1999	Reference	-	Reference	-
2000-2009	0.74 (0.15, 3.75)	0.72	1.25 (0.67, 2.33)	0.48
2010-2017	1.31 (0.32, 5.33)	0.7	0.78 (0.42, 1.47)	0.45
Small intestine				
Sex				
Male	Reference	-	Reference	-
Female	1.06 (0.21, 5.27)	0.94	0.82 (0.33, 2.06)	0.68
Indigenous status				
Non-indigenous	Reference	-	Reference	-
Indigenous	2.90 (0.58, 14.49)	0.2	0.82 (0.24, 2.83)	0.76
Era				
1990-1999	Reference	-	Reference	-
2000-2009	0.88 (0.05, 14.29)	0.93	1.83 (0.38, 8.81)	0.45
2010-2017	3.03 (0.34, 27.42)	0.32	1.69 (0.37, 7.73)	0.5

Table S3. Hazard ratios (HR) and 95% CI (Cox Proportional hazard model) for sex, indigenous status and era by primary sites between two age groups (*n*=1,608).

	(18-50 Years)		(>50 Years)	
	n=298		*n*=1310	
	**HR (95% CI)**	***p*-Value**	**HR (95% CI)**	***p*-Value**
Large intestine				
Sex				
Male	Reference	-	Reference	-
Female	0.82 (0.47, 1.44)	0.5	0.83 (0.67, 1.02)	0.08
Indigenous status				
Non-indigenous	Reference	-	Reference	-
Indigenous	2.22 (1.06, 4.64)	0.03	1.72 (1.19, 2.48)	0.004
Era				
1990-1999	Reference	-	Reference	-
2000-2009	0.73 (0.38, 1.44)	0.37	0.79 (0.61, 1.02)	0.07
2010-2017	0.46 (0.22, 0.98)	0.04	0.60 (0.44, 0.81)	0.0008
Rectum				
Sex				
Male	Reference	-	Reference	-
Female	0.53 (0.25, 1.16)	0.11	0.92 (0.63, 1.35)	0.68
Indigenous status				
Non-indigenous	Reference	-	Reference	-
Indigenous	2.55 (1.13, 5.74)	0.02	1.37 (0.75, 2.49)	0.31
Era				
1990-1999	Reference	-	Reference	-
2000-2009	0.74 (0.33, 1.66)	0.47	0.78 (0.53, 1.14)	0.2
2010-2017	0.47 (0.17, 1.29)	0.15	0.64 (0.39, 1.04)	0.07
Pancreas				
Sex				
Male	Reference	-	Reference	-
Female	1.40 (0.11, 18.24)	0.8	1.10 (0.75, 1.61)	0.64

	(18-50 Years)		(>50 Years)	
	n=298		*n*=1310	
	**HR (95% CI)**	***p*-Value**	**HR (95% CI)**	***p*-Value**
Indigenous status				
Non-indigenous	Reference	-	Reference	-
Indigenous	0.25 (0.01, 8.64)	0.45	1.84 (1.17, 2.89)	0.008
Era				
1990-1999	Reference	-	Reference	-
2000-2009	0.59 (0.03, 13.03)	0.74	0.89 (0.54, 1.48)	0.66
2010-2017	0.21 (0.02, 2.73)	0.23	0.75 (0.46, 1.21)	0.23
Stomach				
Sex				
Male	Reference	-	Reference	-
Female	1.29 (0.13, 12.57)	0.83	0.95 (0.57, 1.60)	0.86
Indigenous status				
Non-indigenous	Reference	-	Reference	-
Indigenous	6.30 (0.75, 53.27)	0.09	1.08 (0.62, 1.88)	0.78
Era				
1990-1999	Reference	-	Reference	-
2000-2009	0.27 (0.03, 2.77)	0.27	1.19 (0.68, 2.07)	0.54
2010-2017	0.88 (0.16, 4.97)	0.88	0.77 (0.42, 1.40)	0.39
Oesophagus				
Sex				
Male	Reference	-	Reference	-
Female	-*		0.59 (0.28, 1.23)	0.16
Indigenous status				
Non-indigenous	Reference	-	Reference	-
Indigenous	-*		3.85 (1.68, 8.82)	0.001
Era				
1990-1999	Reference	-	Reference	-
2000-2009	-*		0.52 (0.27, 1.01)	0.05
2010-2017	-*		0.41 (0.20, 0.84)	0.02
Small intestine				
Sex				
Male	Reference	-	Reference	-
Female	-*		1.49 (0.18, 12.25)	0.71
Indigenous status				
Non-indigenous	Reference	-	Reference	-
Indigenous	-*		5.43 (0.17, 178.26)	0.34
Era				
1990-1999	Reference	-	Reference	-
2000-2009	-*		0.06 (0.00, 1.53)	0.09
2010-2017	-*		0.11 (0.01, 0.83)	0.03

*Reliable estimates of HR (95% CI) cannot be obtained due to small sample size.

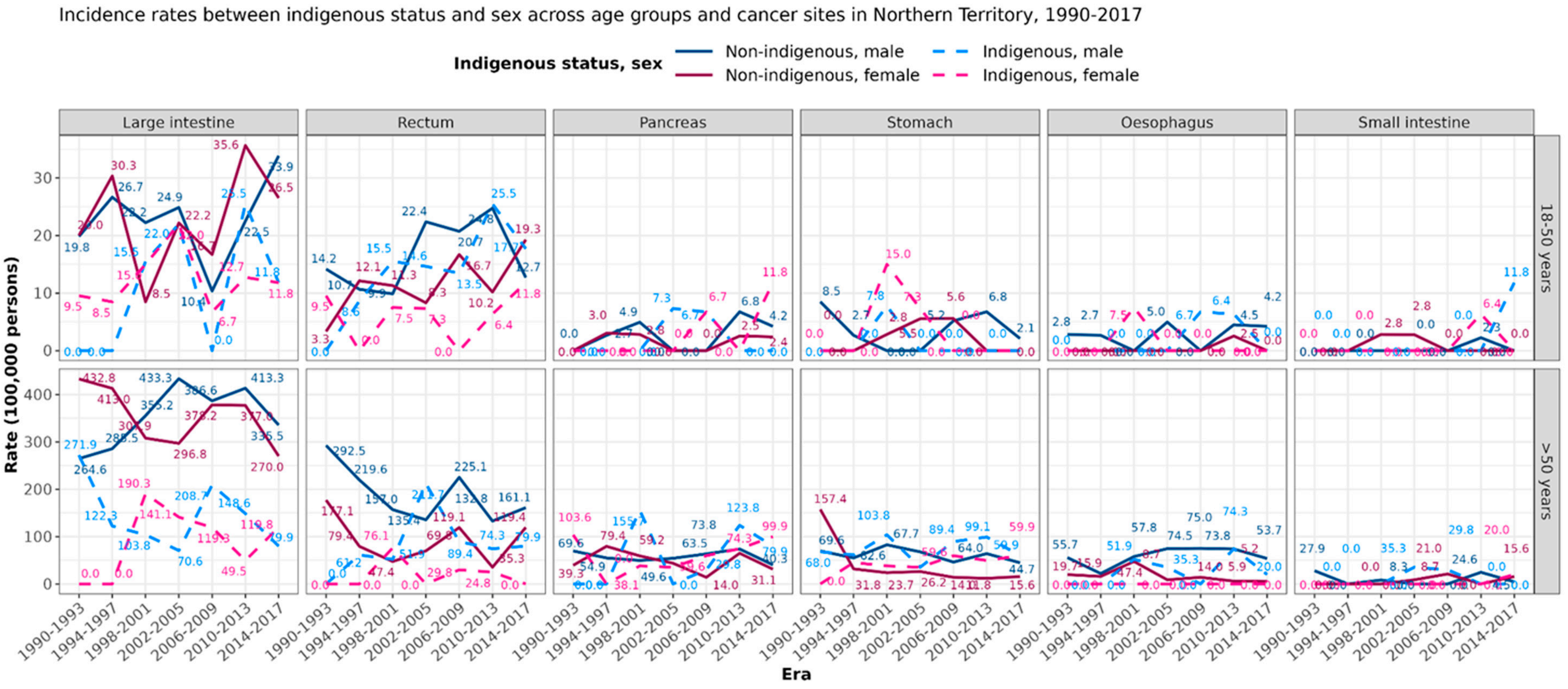

Figure S1. Trend in incidence rates by sex, indigenous status and era between two age groups across cancer sites 1990-2017 (*N*=1,608).

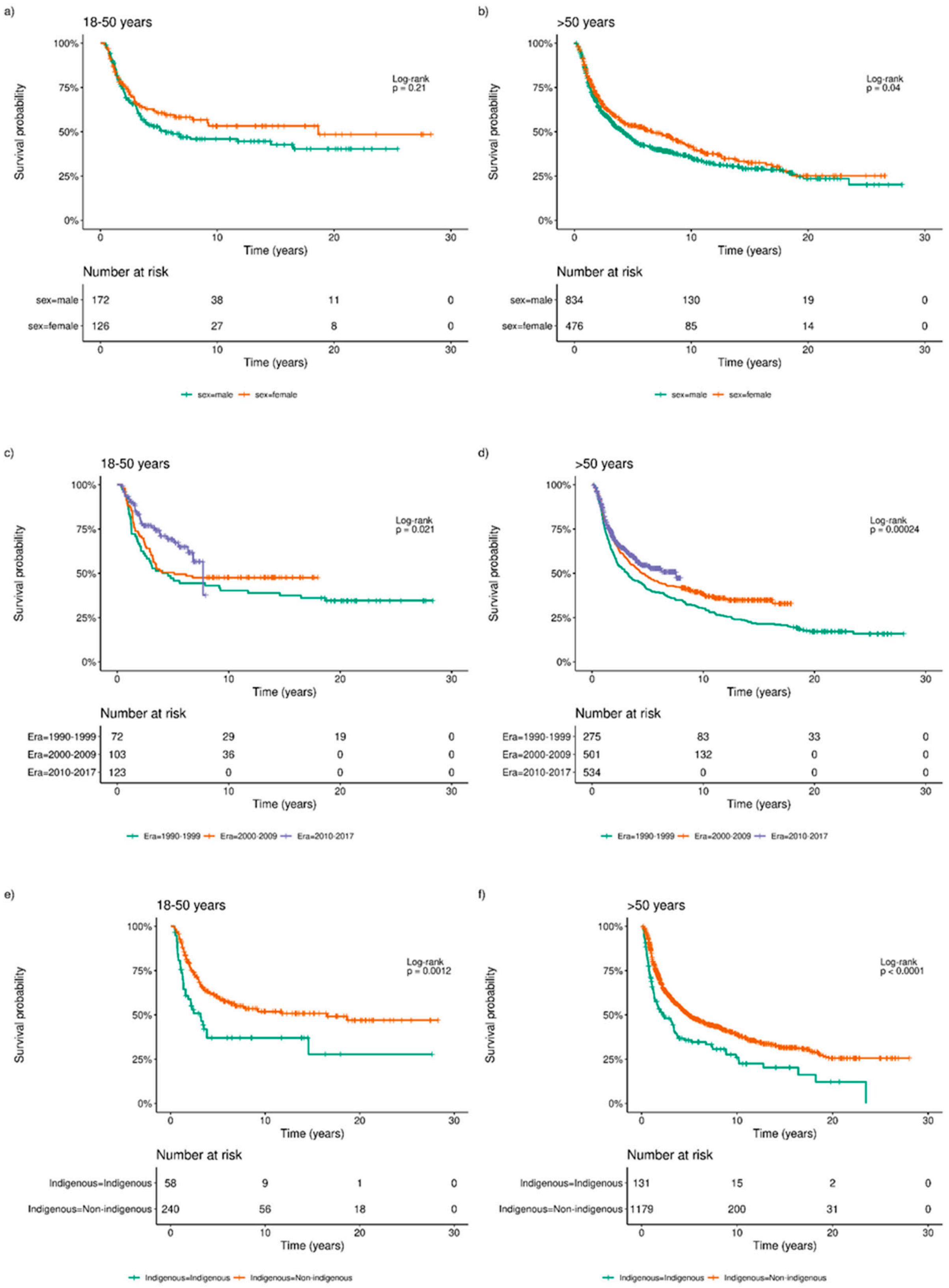
a)
18-50 years
Survival probability
Log-rank
p = 0.21
Time (years)
Number at risk
sex=male 172 38 11 0
sex=female 126 27 8 0
sex=male sex=female
b)
>50 years
Log-rank
p = 0.04
sex=male 834 130 19 0
sex=female 476 85 14 0
c)
18-50 years
Log-rank
p = 0.021
Era=1990-1999 72 29 19 0
Era=2000-2009 103 36 0 0
Era=2010-2017 123 0 0 0
d)
>50 years
Log-rank
p = 0.00024
Era=1990-1999 275 83 33 0
Era=2000-2009 501 132 0 0
Era=2010-2017 534 0 0 0
e)
18-50 years
Log-rank
p = 0.0012
Indigenous=Indigenous 58 9 1 0
Indigenous=Non-indigenous 240 56 18 0
f)
>50 years
Log-rank
p < 0.0001
Indigenous=Indigenous 131 15 2 0
Indigenous=Non-indigenous 1179 200 31 0

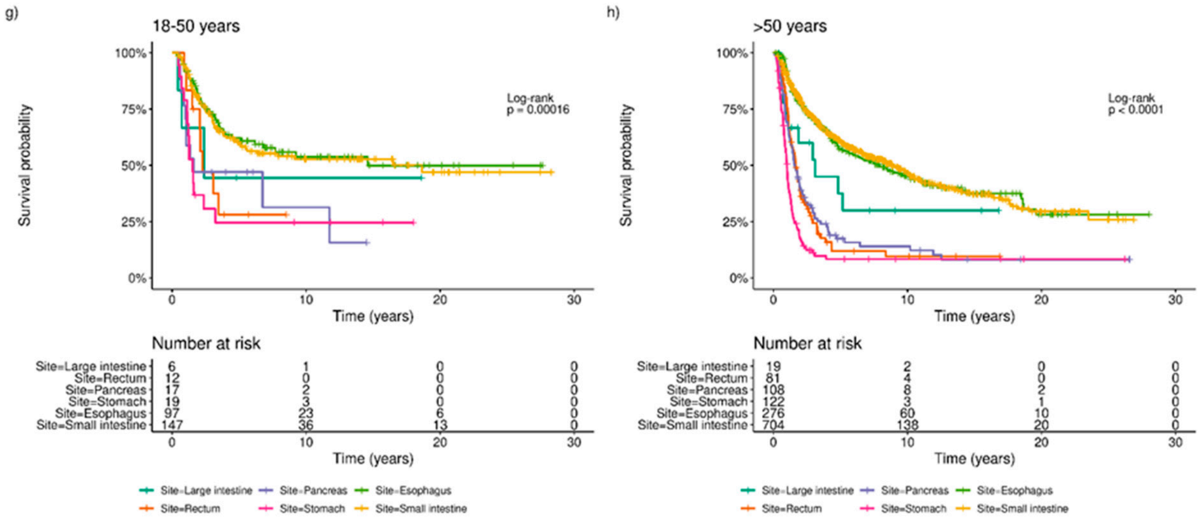

Figure S2. Kaplan-Meier survival curves for sex, era, indigenous status and primary sites between age groups.
